# Immunotherapy based on dendritic cells pulsed with CTPFoxM1 fusion protein protects against the development of hepatocellular carcinoma

**DOI:** 10.18632/oncotarget.10269

**Published:** 2016-06-24

**Authors:** Huiting Su, Bing Li, Lan Zheng, Haixia Wang, Liping Zhang

**Affiliations:** ^1^ Department of Laboratory Medicine, The First Affiliated Hospital, Chongqing Medical University, Chongqing, 400016, China

**Keywords:** cytoplasmic transduction peptide, FoxM1, dendritic cells, hepatocellular carcinoma, immunotherapy

## Abstract

Application of dendritic cells (DCs) pulsed with tumor-associated antigens is considered attractive in immunotherapy for hepatocellular carcinoma (HCC). In order to efficiently prime tumor-associated antigens specific for cytotoxic T lymphocytes (CTLs), it is important that DCs present tumor-associated antigens on MHC class I. MHC class I generally present endogenous antigens expressed in the cytosol. In this study, we developed a new antigen delivery tool based on cross presentation of exogenous antigens in DCs by using cytoplasmic transduction peptide (CTP). CTP protein could transduce FoxM1 tumor antigen into the cytosol of DCs, and CTP-FoxM1 fusion protein could stimulate activation and maturation of DCs. DCs pulsed with CTP-FoxM1 could induce specific CTLs. More importantly, the immunity induced by DCs loaded with CTP-FoxM1 could significantly inhibit tumor growth and metastasis in HCC-bearing mice, which was more potent than that induced by DCs loaded with FoxM1 or CTP, alone. Our results indicate that DCs pulsed with CTP-FoxM1 might be a promising vaccine candidate for HCC therapy and provide new insight into the design of DC-based immunotherapy.

## INTRODUCTION

Hepatocellular carcinoma (HCC) is one of the most prevalent malignant diseases worldwide with a poor prognosis and a high mortality rate [1]. Immunotherapy is an attractive approach for the treatment of HCC, especially for patients at advanced stages [2]. Dendritic cells (DCs) are the most powerful professional antigen-presenting cells (APCs) that can induce antitumor immunity by initiating an antigen-specific cytotoxic T lymphocyte (CTL) response, and application of dendritic cells (DCs) loaded with tumor antigens as anti-tumor vaccines has shown a great potential in therapy and prophylaxis of cancer [3]. DC-based vaccines have been successfully used for the treatment of several cancers including malignant melanoma, HCC, multiple myeloma, acute myeloid leukemia, etc [4–6].

In dendritic cell-based cancer immunotherapy, it is important that DCs should present tumor-associated antigens on MHC class I, which leads to tumor-specific CTL response [7]. However, MHC class I generally present endogenous antigens expressed in the cytosol [8]. Therefore, it is important to develop an approach capable of directly delivering exogenous antigens as endogenous antigens into the cytosol of DCs in DC-based cancer immunotherapy. Cytoplasmic transduction peptide (CTP) is a newly designed transduction peptide which can carry molecules across the cell membrane and locate them into the cytoplasmic compartment [9, 10, 11]. This function of CTP is beneficial for the development of class I-associated CTL vaccines with no side effects on nuclear genetic materials [12, 13]. Our previous study has demonstrated that CTP fusion could transfer bacterial beta-galactosidase into the cytoplasmic compartments in BaF3-BCR/ABL cells and in mouse models [14], suggesting that exogenous antigens fused to CTP could be recognized as endogenous antigens when delivered into the cytosol, facilitating the use of CTP fusion protein transduction as a promising antigen delivery system in DC-based cancer immunotherapy.

The Forkhead box protein M1 (FoxM1) belongs to a large family of forkhead box (Fox) transcription factors. FoxM1 expression is mainly detected in the progenitor and regenerating tissues, and it is overexpressed in various human malignancies including liver, prostate, breast, lung, colon, pancreas, ovary, etc [15]. Overexpression of FoxM1 in various tumors indicates a strong dependence of the tumor cells on FoxM1 expression because of an integral role of FoxM1 in tumorigenesis [15–19]. Previous studies have shown that FoxM1 was essential for development of HCC, and overexpression of FoxM1 was associated with aggressive tumor features and poor prognosis [20]. In fact, FoxM1 could induce an epithelial-mesenchymal-like transition phenotype in HCC cells, increase cell migration, and induce premetastatic niche at the distal organ of metastasis [21, 22]. Down-regulation of FoxM1 could suppress the proliferation of HCC cells and inhibit HCC growth [23]. These studies suggest that FoxM1 plays an important role in the development of HCC, which is a new therapy target for HCC therapy.

In this study, we created a CTP-FoxM1 fusion protein and investigated its anti-tumor activity against HCC elicited by DCs pulsed with CTP-FoxM1. We found that CTP-FoxM1could induce activation and maturation of DCs. DCs loaded with CTP-FoxM1 could induce potent FoxM1-specific T cell immune responses. More importantly, the immunity induced by CTP-FoxM1-loaded DCs could significantly inhibit tumor growth and metastasis in HCC-bearing mice, which was more potent than that induced by DCs loaded with FoxM1 or CTP, alone.

## RESULTS

### Purification and characterization of CTP-FoxM1 fusion protein

**Figure 1:**
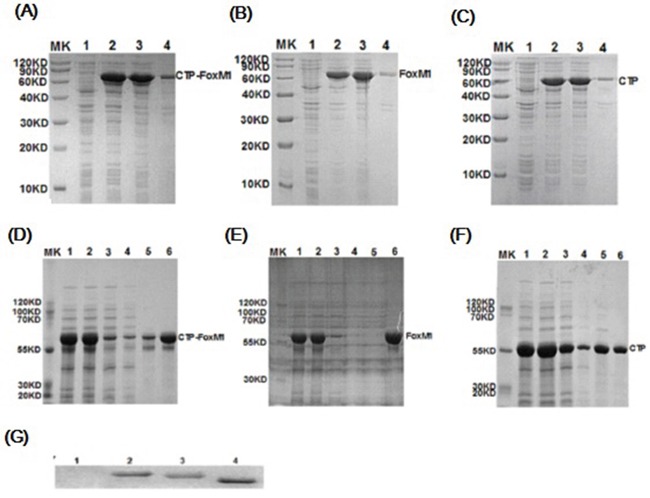
Expression, purification and characterization of recombinant proteins **A~C.** Induced expression of CTP-FoxM1, FoxM1, CTP protein. MK: prestained protein; 1: non-induced crude; 2: induced crude; 3: supernatant of lysate; 4: precipitation of lysate; **D~F.** Purification of CTP-FoxM1, FoxM1, CTP protein. MK: prestained protein; 1: IPTG induced culture; 2: supernatant of centrifugal IPTG induced culture; 3: column flow-through; 4: binding buffer; 5: imidazole wash buffer; 6: 500 mM imidazole elution buffer; **G.** Identification of CTP-FoxM1 fusion protein. 1: prestained protein; 2: CTP-FoxM1 fusion protein; 3: FoxM1 protein; 4: CTP protein

Prokaryotic expression vectors including pcold-TF-CTP-FoxM1, pcold-TF-CTP and pcold-TF-FoxM1 were successfully constructed, and no mutation was found by sequencing (data not shown). The corresponding CTP-FoxM1 and control CTP, FoxM1 proteins were successfully expressed by isopropyl β-D-1-thiogalactopyranoside (IPTG) induction at a final concentration of 1 mM at 37°C for 3h and purified by Ni2+-affinity column (Figure 1A~1C). The predicted molecular mass of recombinant CTP-FoxM1 protein is 59.6 kDa. These recombinant proteins were identified by SDS–PAGE gel and subsequently stained with Coomassie brilliant blue staining, which demonstrated that these recombinant proteins have been purified to near homogeneity and approximately 95% purity (Figure 1D~1F). Moreover, the expression and purification of the CTP-FoxM1 protein and control protein CTP or FoxM1 was subsequently immunologically confirmed by western blot analysis using anti-his specific antibody (Figure 1G). Moreover, the activity of LPS in protein preparation was less than 25 EU/mg (data not shown).

### CTP fusion could transduce FoxM1 into the cytosol of DCs

**Figure 2:**
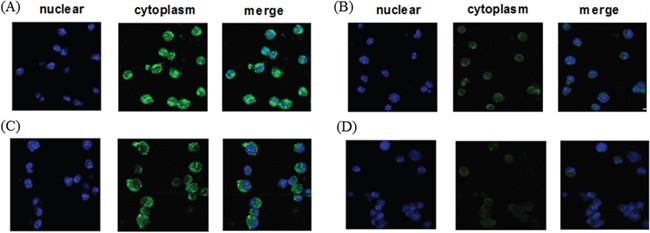
Cytoplasmic localization of recombinant protein in DCs DCs treated with **A.** CTP-FoxM1, **B.** FoxM1, **C.** CTP, **D.** PBS were fixed and stained with anti-his antibody and FITC-labeled rabbit anti-mouse IgG. Confocal microscopic analysis was then performed to evaluate the localization of recombinant proteins. Four consecutive confocal images were merged for each picture.

In an attempt to confirm whether exogenous CTP-FoxM1 fusion protein was localized in cytoplasm, DCs were incubated with recombinant CTP-FoxM1, CTP or FoxM1 proteins. Subsequently, these cells were fixed and stained with antibodies and DAPI dihydrochloride. We demonstrated that the majority of the CTP-FoxM1-specific fluorescent (FITC) signals (green) were detected in the cytoplasm, and they were clearly separated from the nucleus-specific DAPI signals (blue) in the DCs incubated with CTP-FoxM1 (Figure 2). These results indicated that CTP-FoxM1 protein was successfully localized into the cytoplasmic compartment of the DCs.

### CTP-FoxM1 could induce maturation and activation of DCs

**Figure 3:**
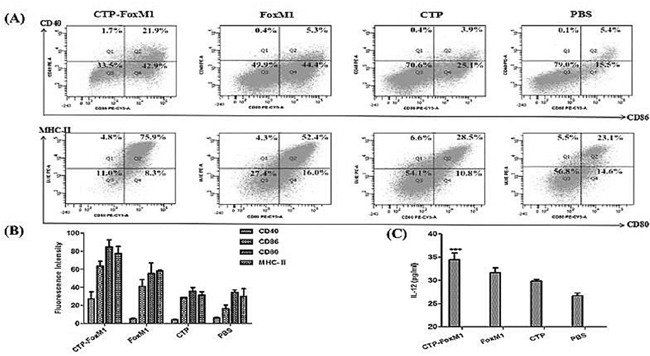
CTP-FoxM1 induced maturation and activation in DCs **A.** Flow cytometric analysis of cell surface molecules on DCs. DCs were treated with 1 μg/ml of CTP-FoxM1, FoxM1, CTP or PBS for 48 h, and DCs were then stained with CD11c antibodies labeled with FITC, MHC-II and CD80 antibodies labeled with PE-Cy5, CD40 and CD80 antibodies labeled with PE, respectively. **B.** The expression of MHC-II, CD40, CD80, and CD86 was increased in comparison to non-stimulated DCs and the mean fluorescence intensity was presented. **C.** IL-12 release in the supernatants of DCs. Mice monocyte-derived DCs were incubated with PBS, CTP-FoxM1, FoxM1, and CTP for 48 h. The supernatants were collected and assessed for the level of IL-12 by ELISA. The data shown are the mean of triplicate experiments; the bars represent the mean ± SD; *** P<0.01 when compared with DCs stimulated by FoxM1, CTP or PBS. n = 5 mice/group.

After 5 days in culture with GM-CSF and IL-4, immature DCs were generated by differentiation of mononuclear cells. Recombinant CTP-FoxM1, CTP, or FoxM1 at 1 ug/mL of concentration was finally chosen to stimulate DC for 48 h according to CCK-8 experiments (Table 1). Then, the important surface molecules of DCs were detected by flow cytometry. Based on FACS analysis, CTP-FoxM1 protein induced a significant increase in expression of CD40 (27.2±7.75%), CD86 (63.47±5.23%), CD80 (84.73±7.81%), and MHC- (77.37±8.04%) compared with PBS, FoxM1, or CTP, alone (Figure 3A~3B). Besides, the culture supernatants were also collected and then measured by ELISA. As a result, the release of IL-12 was 34.43±1.43 pg/ml in the medium of DCs loaded with CTP-FoxM1 protein, which was significantly higher than that from DCs loaded with PBS, FoxM1, or CTP alone (Figure 3C)[24].

**Table 1:** Cell survival rate of DCs loaded with CTP-FoxM1, FoxM1, CTP

concentration(ug/ml)	CTP-FoxM1	FoxM1	CTP
0.2	92.5%±5.5%	97.73±0.5%	92.13±2.73%**
0.5	86.90±4.54%**	94.57±3.06%	73.17±6.73%**
1	80.93±8.36%**	87.23±6.05%**	65.23±1.47%**
2	70.13±5.38%**	74.77±3.42%**	59.23±0.15%**
4	62.97±4.06%**	71.30±2.62%**	53.70±1.22%**

** P<0.01 compared with PBS control group (Cell survival rate was 100%)

### CTP-FoxM1 activated DCs to generate antigen-specific CD8+ T cell responses

**Figure 4:**
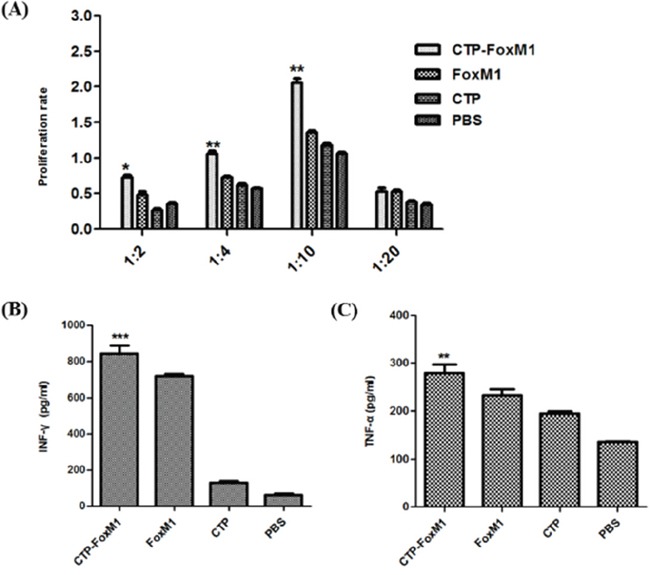
DCs activated by recombinant antigens generated antigen-specific T cell response **A.** Effects of different DC vaccination on T lymphocytes proliferation. T lymphocytes derived from C57BL/6 mice were co-cultured with PBS-DC, CTP-FoxM1-DC, FoxM1-DC, and CTP-DC for three days. Reactive T cells and stimulated cells were in ratio of 1:2, 1:4, 1:10, and 1:20. **B.** Levels of IFN-γ from CD8+T cell co-cultured with DCs pulsed with different antigens. Each group of DCs was incubated with 1ug/ml CTP-FoxM1, FoxM1, CTP, or PBS for 48h. CD8+T cells derived from C57BL/6 mice spleen were co-cultured with different group of DCs for 48h. The supernatant were harvested and IFN-γ in the supernatant was analyzed by ELISA. *P<0.05, **P<0.01, ***, P<0.001 when compared with DCs pulsed with PBS, FoxM1, or CTP. The data shown are the mean of triplicate experiments; the bars represent the mean ± SD. n = 5 mice/group. **C.** Levels of TNF-α from CD8+T cell co-cultured with DCs pulsed with different antigens. TNF-α in the supernatant was also analyzed by ELISA. *P<0.05, **P<0.01, ***, P<0.001 when compared with DCs pulsed with PBS, FoxM1, or CTP. The data shown are the mean of triplicate experiments; the bars represent the mean ± SD. n = 5 mice/group.

The effect of CTP-FoxM1-DCs on the proliferation of T lymphocyte was evaluated using CCK-8 assays. As shown in Figure 4A, DCs pulsed with CTP-FoxM1 induced significantly higher proliferation of splenocytes than those in other groups. CTP-FoxM1-loaded DCs had more efficient effect on stimulating the proliferation of T cells at equal stimulator ratios (10:1) when compared with those in other controls.

CD8^+^T cells derived from C57BL/6 mice were further negatively selected using the CD8^+^ T Cell Isolation Kit and measured by the FCM, and its purity was up to 90% (data not shown). In order to determine whether CTP-FoxM1-loaded DCs could induce antigen-specific CD8+ T cell responses, we measured the production of cytokines IFN-γ and TNF-α in co-culture supernatants as surrogate markers for FoxM1-mediated activation of CD8^+^ T cells. The supernatants of CD8^+^T cells co-cultured with CTP-FoxM1-loaded DCs exhibited higher levels of IFN-γ and TNF-α compared with those in other groups (Figure 4B~4C). Thus, CTP-FoxM1 activated DCs to generate antigen-specific CD8+ T cell responses, which may have anti-cancer immune responses.

### DCs pulsed with CTP-FoxM1 induced CTL effects on HCC cells

**Figure 5:**
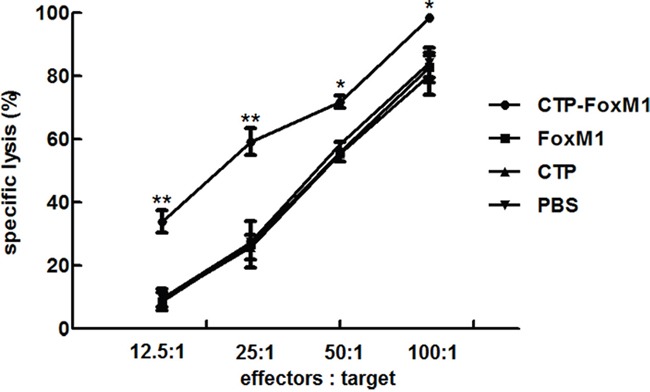
Level of CTLs induced by DCs pulsed with different antigens Each group of mice was administrated for three times at weekly interval with DCs pulsed with CTP-FoxM1, FoxM1, CTP, or PBS. One week after the last administration, splenocytes isolated from immunized mice from each group mentioned above were co-cultured with Hepa1-6 cell. Different effector/target cell ratios were mixed for 24 h. The lysis of target cells was determined by LDH release. The experiments were performed in triplicate, and the bars represent the mean ±SD; *P<0.05; ** P<0.01 when compared with DCs pulsed with PBS, FoxM1, or CTP; n = 5 mice/group.

To evaluate the functional effects of CTL generated by DCs loaded with protein antigens, we carried out *in vitro* cytotoxicity assays using lymphocytes isolated from C57BL/6 mice injected with DCs loaded with CTP-FoxM1-DC, FoxM1-DC, CTP-DC, or PBS. LDH release assay was used to evaluate the cytolytic activity of effector cells. FoxM1 was highly expressed in Hepa1-6 hepatoma cell lines regarded as target cells (data not shown). In the group of DCs pulsed with CTP-FoxM1, CTL activity at the E/T ratios of 12.5:1, 25.0:1, 50.0:1and 100.0:1, was (33.89±3.61)%, (59.21±4.26)%, (71.83±1.94)% and (98.49±0.77)%, respectively, which was significantly higher compared with those in other groups (Figure 5). These results demonstrated that CTP-FoxM1-loaded DCs could induce significant CTL activity against Hepa1-6 cells.

### DCs pulsed with CTP-FoxM1 induced therapeutic anti-tumor effects in mice

**Figure 6:**
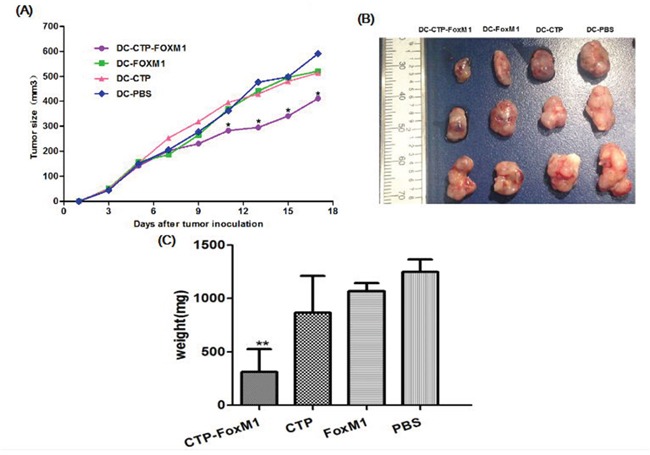
Effects of immunization with DCs pulsed with antigens on tumor size in the treatment of established tumor models Mice were inoculated subcutaneously with Heap 1-6 tumor cells (day 0). On days 7 and 14, mice were injected with DCs loaded with different Ag mixtures, shown in the figure. The tumor volume in all vaccination groups was measured from day 1 at 2-day intervals for 19 days. **A.** At day 11, 13, 15, 17, the average tumor volume in tumor-bearing mice immunized with DCs pulsed with CTP was significantly smaller than those in mice immunized with DCs pulsed with FoxM1, CTP or PBS (*P<0.05). **B.** The image of tumor tissue masses **C.** the mean weight of tumor masses. n = 10 mice/group.

We then evaluated whether DCs pulsed with CTP-FoxM1 could suppress tumor growth in HCC-bearing C57BL/6 mice. C57BL/6 mice were inoculated subcutaneously with Hepa1-6 cells in the right flank at day 0. At day 7, and 14, mice were immunized subcutaneously with DCs pulsed with CTP-FoxM1, CTP, FoxM1 or PBS in the left flank. Tumor-bearing mice immunized with DCs pulsed with CTP-FoxM1 showed a significantly slower tumor growth (Figure 6A) and a dramatic reduction in tumor size (Figure 6B), Moreover, the weight of tumor mass was also significantly lower in the group immunized with DCs pulsed with CTP-FoxM1 (Figure 6C). Hence, these results demonstrated that CTP-FoxM1-loaded DCs could induce anti-tumor immune responses in HCC mouse model.

### DCs pulsed with CTP-FoxM1 induced prophylactic anti-tumor effects in mice

**Figure 7:**
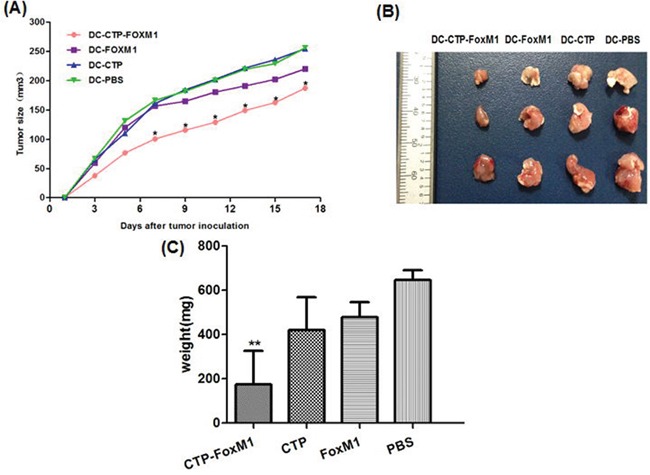
DCs pulsed with CTP-FoxM1 induced prophylactic anti-tumor effects After injection with DCs loaded with CTP-FoxM1, FoxM1, CTP or PBS once a week for three times, mice were inoculated subcutaneously with Heap 1-6 tumor cells (day 0). The tumor volume in all vaccination groups was measured from day 1 at 2-day intervals for 19 days. **A.** At day 7, 9, 11, 13, 15, 17 the average tumor volume in tumor-bearing mice vaccinated with DCs pulsed with CTP-foxM1 was significantly smaller than other groups (*P<0.05). **B.** The image of tumor tissue masses **C.** the mean weight of tumor mass. n = 10 mice/group.

**Figure 8:**
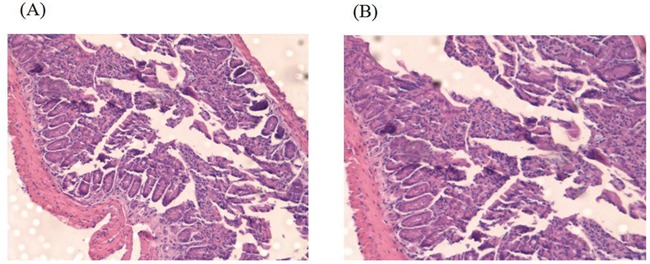
HE staining of Small intestine **A.** Small intestine structure of C57BL/6 mice of CTP-FoxM1 group by HE staining; **B.** Small intestine structure of C57BL/6 mice of untreated group by HE staining.

We further set out to evaluate the potential of CTP-FoxM1-loaded DCs in clearing tumors. C57BL/6 mice were vaccinated with DCs pulsed with CTP-FoxM1, CTP, FoxM1 or PBS once every week for three times. These mice were then challenged using subcutaneous injection with Hepa 1-6 cells after the last immunization. They were observed for 19 days after tumor challenge. Notably, vaccination with DCs pulsed with CTP-FoxM1 provided more efficient tumor suppression in tumor growth and size compared with other groups vaccinated with DCs pulsed with CTP, FoxM1 or PBS (Figure 7A). After 3 weeks, the tumors were excised from the animals. Results indicated that the mean tumor weight of the CTP-FoxM1-DCs group was less than those of the other groups (Figure 7B~7C). Hematoxylin-eosin (HE) staining showed that there was no metastasis and observed injury in the small intestine of mice (Figure 8). These results demonstrated that vaccination with DCs pulsed with CTP-FoxM1 could decelerate tumor progression.

## DISCUSSION

Therapeutic vaccination with DCs pulsed with tumor-associated antigens represents an attractive approach for HCC treatment [25]. In DC-based cancer immunotherapy, DCs should present peptides derived from tumor-associated antigens on MHC class I and then activate tumor-specific CTL response. MHC class I generally present endogenous antigens expressed in the cytosol [8]. In this study, we developed a new way capable of directly delivering exogenous recombinant FoxM1 tumor antigens by fusion with CTP into the cytosol of DCs, indicating that exogenous tumor antigens can be recognized as endogenous antigens when delivered into the cytosol of DCs by CTP. In this study, immunization with DCs pulsed with CTP-FoxM1 fusion proteins efficiently induced FoxM1-specific CTL response and protected against the development of HCC. Our data thus provided a novel immunotherapeutic approach for the treatment of HCC.

Recently, various ways of delivering tumor antigens into DCs *in vitro* and *in vivo* have been developed. These include microbial components loaded with tumor antigens, antigen transfer mediated by lentivirus vector, HSP–‘antigen’ complex-mediated cross-presentation and Virus-like particles (VLP) delivery system for proteins, etc [26–28]. CTP is a newly designed transduction peptide that carries molecules across the cell membrane with a preference to localize in the cytoplasmic compartment, providing promising therapeutic opportunities for the treatment of various diseases caused by cytoplasmic functional molecules. CTP has a strong cell-penetrating property, and can deliver CTP-fused antigens into the cytoplasm of cells [12–14]. Here we reported that CTP-fused antigens can successfully locate into the cytoplasm of DCs. DCs pulsed with CTP-fused tumor-associated antigens cloud elicit potent antigen-specific CTL response when compared with DCs pulsed with antigen alone. Our data firstly suggest that CTP is a promising tumor-associated antigen delivery system in DC-based HCC immunotherapy.

Regarding the selection of HCC-associated tumor antigens, we have identified FoxM1 as a potential target for immunotherapy. Several important features of this target molecule are described as follows. Firstly, we have found that FoxM1 was highly expressed in 70% of adult HCC patients and HCC cell lines (unpublished data). Secondly, FoxM1 is not expressed, or is expressed at very low levels in normal tissues [21]. Thirdly, FoxM1 plays an essential role in HCC cell migration, invasion, as well as liver cancer progression and in cancer cells with stem cell features [15, 22]. Fourthly, clinicopathologic studies suggest that FoxM1 expression correlated with poorly-differentiated HCC tumors with intrahepatic metastasis, which is a leading cause of post-surgical recurrence and low survival rate [20, 29]. Finally, silencing of FoxM1 expression could inhibit human hepatocellular carcinoma growth [30], and FoxM1 has been reported an underlying therapeutic target because it can be presented to cell surface by tumor cells [31]. Therefore, FoxM1 is considered as a novel therapeutic target for HCC drug therapy.

Here we prepared a fusion protein consisting of FoxM1 and CTP to improve the anti-tumor effects of DC-based immunotherapy against HCC, and we observed that CTP-FoxM1 fusion protein, but not FoxM1 or CTP, could significantly up-regulate the expression of co-stimulatory molecules including CD40, CD80 and CD86 on the surface of DCs. Moreover, DCs loaded with CTP-FoxM1 fusion protein produced a significantly higher level of IL-12 compared with that of DCs loaded with FoxM1 or CTL, alone. The ability of CTP-FoxM1 fusion protein to stimulate DC maturation was unlikely attributed to the contaminant of endotoxin, because the activity of LPS in CTP-FoxM1 fusion protein was less than 25 EU/mg, and heat-inactivated proteins could not induce the expression of co-stimulatory molecules on the surface of DCs (data not shown). Therefore, CTP-FoxM1 fusion protein has the ability to induce maturation and activation in DCs.

In our study, the purified CTP-FoxM1 fusion protein was mainly localized into the cytoplasmic compartment of DCs, while FoxM1 was scarcely located into the cytoplasm of DCs, suggesting that DCs could present cytoplasmic location of CTP-FoxM1 as endogenous antigen. Since CD8+ T cells are critical for inhibiting tumor growth [32], we studied the role of DCs pulsed with CTP-FoxM1 in regulation of CD8+ T cells. CD8^+^T cells co-cultured with DCs pulsed with CTP-FoxM1 produced significantly higher levels of IFN-γ and TNF-α compared with those co-cultured with DCs pulsed with FoxM1 or CTP, alone, suggesting that DCs loaded with CTP-FoxM1 fusion protein cloud exert more efficient function of cross-presentation compared with FoxM1-loaded DCs. Moreover, DCs pulsed with CTP-FoxM1 could induce more potent CTL activity against Hepa1-6 cells compared with DCs pulsed with CTP or FoxM1, alone. In hepatic carcinoma mouse models, immunization of DCs pulsed with CTP-FoxM1could elicit both therapeutic and prophylactic anti-tumor effects against the development of HCC. However, our CTP-FoxM1 fusion protein was purified from prokaryotic expression vector. Future studies should take into account the use of CTP-FoxM1 from eukaryotic expression systems and the immunization route in order to optimize DC-based immunotherapy against HCC.

In conclusion, the current study identified that DCs pulsed with CTP-FoxM1 could significantly inhibit tumor growth and metastasis in HCC-bearing animals. We propose that this type of DC-based immunotherapy may be applicable to breast carcinoma, pulmonary cancer, and other types of tumors expressing FoxM1 [15–19].

## MATERIALS AND METHODS

### Animals and cell lines

Female C57BL/6 mice (6-8 weeks old) were purchased from the animal experimental center of Chongqing medical university. All mice were maintained under specific pathogen-free conditions. All experiments were carried out according to the National Institutes of Health Guide for Care and Use of Laboratory Animals and were approved by the Ethics Committee of the first affiliated hospital of Chongqing medical university. The tumor size of 20 mm was used as a surrogate endpoint of survival, and mice were in deep anesthesia using pentobarbital sodium and euthanized by high concentration of CO2 inhalation. The HCC cell line Hepa1-6 expressing FoxM1 was obtained from Wuhan Type Culture Collection and cultured in DMEM high glucose with10% FBS, 100 U/ml penicillin, 100 mg/ml streptomycin (Sigma, St. Louis, MO, USA).

### Preparation of recombinant proteins

According to the cDNA gene sequence (cDNA, NM-008021) of C57BL/6 mice in GenBank, we selected the key sequence of FoxM1 antigen epitope, GGT CTG ATG GAA CTG AAT ACC ACA CCGCTG, and connected 4 polymers in series through DNA ligase to enhance the activity of antigen. Then the full-length gene sequence was inserted into prokaryotic expression vector pCold-TF, designated as pCold-TF-CTP-FoxM1. The pCold-TF-CTP-FoxM1 was connected with His-tag to load on Ni2+-affinity column in order to purify expressed protein. The recombinant plasmid was synthesized by Sangon Biological Engineering Technology and Service Co. (Shanghai, China). The plasmid was then transformed into *E. coli* BL21 (DE3) and recombinant CTP-FoxM1 was expressed under IPTG induction. To purify CTP-FoxM1, the induced bacteria were harvested and dissociated by Ultrasonic Disruptor. After centrifugation, the supernatant was loaded successively onto Ni2+-affinity column. The collected eluate was desalted and removed imidazole by Ultrafiltration cup. The purified proteins were verified by Western blotting using anti-his tag specific mAb (CST, Boston, USA), and quantified by the Nandrop 2000. In a similar way, both FoxM1 and CTP was expressed, purified, ultrafiltered and verified. The LPS in protein preparations was determined with the Limulus amebocyte lysate assay (Zhanjiang Bokang Marine Biological Co., Ltd., China) according to the manufacturer's instructions.

### Separation and cultivation of DCs

Bone marrow-derived DCs (BMDCs) were generated according to a previously described procedure [33]. Briefly, bone marrow from the femurs and tibias of female C57BL/6 mice was grown in RPMI 1640 with 10% FBS, 100 U/ml penicillin, 100 mg/ml streptomycin, and 20 ng/ml GM-CSF (R&D Systems), 10ng/ml IL-4 (R&D Systems) after the red blood cells were lysed at 37°C in a humidified CO2 incubator. Cultures were initiated by placing 2×10^7^ bone marrow cells in 24 ml of medium onto 6-well culture dishes. On day 3, the non-adherent cells were gently removed from 6-well plates, and the loosely adherent cells were cultured in medium with fresh medium with GM-CSF and IL-4. On day 5, another 24 ml of fresh medium with GM-CSF and IL-4 were replaced. Seven days later, BMDCs were collected and incubated with the serum of rat at room temperature for 30 min, and then stained with anti-mouse CD11c-FITC antibody (Ebioscience, CA, USA) kept in dark place at 4°C for 30 min. After washing stained BMDCs with PBS twice, it was analyzed by FACSCalibur™ flowcytometer (Becton Dickinson, Franklin Lakes, NJ, USA). Acquired data were analyzed using FlowJo software (Tree Star, Ashland, OR). The non-adherent and loosely adherent DCs were harvested by vigorous washing. These cells generally consisted of 50-90% DC as assessed by morphology and phenotype.

### Phenotypic analysis of DCs

DCs from Bone marrow were incubated with CTP-FoxM1 at 1ug/mL in 10% fetal bovine serum RMPI 1640 at 37°C for 48h. Survival rate of DCs was detected by Cell Counting Kit-8. Intracellular localization analysis by immunocytochemistry and visualized by confocal microscopy (LEICA Lasertech GmbH, Heidelberg, Germany). The DCs maturation markers including CD40, CD80, CD86, MHC-II were determined by flow cytometry using a FACSCalibur™ flow cytometer (Becton Dickinson, Franklin Lakes, NJ, USA). The following monoclonal antibodies were used: i) fluorescein isothiocyanate-conjugated mouse antihuman IgG2a isotype control; ii) phycoerythrin -conjugated mouse antihuman IgG1 isotype control; iii) phycoerythrin-CY_5_conjugated mouse antihuman IgG1 isotype control; iiii) anti-CD40, anti-CD80, anti-CD86 anti- MHC-II (eBioScience, San Diego, CA, USA). The culture supernatants were also collected, and the level of IL-12 were quantified using commercial ELISA kits purchased from Xinbosheng according to manufacturer's instructions.

### Purification of CD8+ T cells from the spleen of C57BL/6 mice

CD8^+^T cells from C57BL/6 mice spleen were negatively selected using the CD8a+ T Cell Isolation Kit (MACS). Mouse CD8+ T cells were isolated by depletion of non-target cells which were indirectly magnetically labeled with a cocktail of biotin conjugated monoclonal antibodies. The purity of the resulting CD8^+^ T populations was examined by flow cytometry with CD8-PE antibody and it was found to be consistently >90%.

### *In vitro* cross-presentation

DCs were incubated with 1 μg/ml CTP-FoxM1 fusion protein in RPMI-1640 with 10%FBS for 48 h. Similarly, DCs were incubated with 1 μg/ml CTP, FoxM1 protein and PBS in RPMI-1640 with 10%FBS for 48 h as the control. The incubated DCs were collected and then washed three times with PBS. These cells were adjusted into a concentration of 2×10^5^/mL by RPMI-1640 with 10% FBS. 0.1ml of DCs suspension was co-cultured with 1×10^5^ CD8+ T cells which were isolated from C57BL/6 mice spleens using a MACS CD8+T-cell isolation kit (Miltenyi, Biotec) in complete medium in 96-well round bottom plates. After 48 hours of co-cultivation, the supernatants were collected, and the levels of IFN-γ and TNF-α were quantified using commercial ELISA kits purchased from Xinbosheng according to manufacturer's instructions.

### Lymphocyte proliferation assay

As reactive cells, the isolated spleen cells after lysis of red cells were adjusted into a cell concentration of 2×10^5^/ml. DCs loaded with 1 ug/ml concentration of CTP-FoxM1 protein were collected, mitomycin C to a final concentration of 25ug/ml was added, and then bathed in 37°C for 20 min, and then washed 3 times with PBS, regarding as stimulating cells. These stimulating cells were adjusted into a concentration of 4×10^6^/mL by RPMI-1640 with 10% FBS. Each well of 96-well culture plate was filled with reactive T cells and stimulating cells at the ratio of 1:2, 1:4, 1:10, 1:20 (reactive cells to stimulating cells). The volume of medium in each well was 200 ul. There were six wells in each group. The cells were cultured under 37°C, in 5% CO2 for 72h. 3 h before completion, CCK-8 was added in all wells, cut-off OD value was measured at 492 nm using a microplate reader (Tecan, Austria). T cell proliferation rate = (experiment group OD- machine background OD)/(negative control OD- machine background OD), and presented as mean ± SD.

### CTL assay

Cytotoxic function of splenocytes (activated T lymphocytes) from mice (5 mice/group) injected with CTP-FoxM1-DCs vaccine was determined by LDH (Roche) cytotoxicity assay. All steps were performed following the manufacturer's instructions. Briefly, the splenocytes (activated T lymphocytes) were derived from immunized C57BL/6 mice. These splenocytes were regarded as the effector cells. The expression of FoxM1 in the hepa1-6 hepatoma cell lines were verified by Western blotting using anti-FoxM1 specific mAb. The effector cells (E) were cocultured with 3×10^4^ cells/well of target cells (T), hepa1-6 hepatoma cell lines, at the E:T ratios of 12.5:1, 25:1, 50:1, and 100:1 in 96-well culture plates at a total volume of 200ul/well for 24 hours at 37°C, 5% CO2. Lymphocytes from unimmunized mice and target cells cultured with medium alone were used as controls. The spontaneous release of LDH by target cells or effector cells was assayed by incubation of target cells in the absence of effector cells and vice versa, the maximum release of LDH was determined by incubation of the target cells in lysis solution. The supernatants were measured by LDH assay and absorbance was detected at 492 nm using a microplate reader (Tecan, Austria). The percentage of cytotoxicity at each effector-to-target cell ratio was calculated as below formula: Cytotoxicity (%) = [A492nm(experimental) − A492nm (effector spontaneous) − A492nm (target spontaneous)] × 100/[A492nm(target maximum)− A492nm(target spontaneous)].

### Protection against HCC generated by DCs loaded with recombinant antigens

Tumors were generated through subcutaneously injection with 3×10^6^ Hepa1-6 cells in 0.1 mL of PBS into the right flank of each C57BL/6 mouse. The mice were divided into four groups (10 mice per group): (1) CTP-FOXM1 group, which was treated with 1ug/ml concentration of CTP-FOXM1-DCs (1×10^6^ cells per mouse) after 7 days of inoculating Hepa1-6 cells; (2) CTP group, mice were subcutaneously injected with 1×10^6^ DCs activated by CTP after 7 days of inoculating Hepa1-6 cells; (3) FOXM1 group, mice were subcutaneously injected with 1×10^6^ DCs activated by FOXM1 after 7 days of inoculating Hepa1-6 cells; (4) PBS group, mice were subcutaneously injected with 1×10^6^ DCs activated by PBS as the control. The mice were treated once a week for 2 weeks. In another study, mice were divided into four groups as described above to investigate whether CTP-FoxM1-DCs vaccine had an immunoprophylaxis role in HCC of mice. First of all, the mice were subcutaneously injected with DCs loaded with antigens (recombinant CTP-FoxM1, CTP, FoxM1) into the right flank once a week for 3 weeks. And then mice were subcutaneously injected with 3×10^6^Hepa1-6 cells into the contralateral flank after the third immunization. The development of tumor was observed and the perpendicular diameters of individual tumor were monitored every 2 days. The tumor volume was determined as (short diameter) ^2^×long diameter×0.50. Animals were killed when tumor size exceeded 20 mm and the mean weight of tumors mass was measured after the dissection of tumors. The small intestines were obtained and stained by HE after mice were killed.

### Statistical analysis

The results were expressed as means ± SD. The statistical significance of difference between the groups was determined by applying the two independent sample *t*-test after each group had been tested with equal variance and Fisher's exact probability test. The statistical significance of differences in more than 2 groups was determined by applying one-way ANOVA. p<0.05 was considered significant. Significant differences are noted as * p<0.05, ** p<0.01, and *** p<0.001.
